# Conductive Hydrogel Inspires Neutrophil Extracellular Traps to Combat Bacterial Infections in Wounds

**DOI:** 10.1021/acsnano.4c14487

**Published:** 2025-03-03

**Authors:** Lizhi OuYang, Ze Lin, Xi He, Jiaqi Sun, Jiewen Liao, Yuheng Liao, Xudong Xie, Weixian Hu, Ruiyin Zeng, Ranyang Tao, Mengfei Liu, Yun Sun, Bobin Mi, Guohui Liu

**Affiliations:** † Department of Orthopedics, Union Hospital, Tongji Medical College, Huazhong University of Science and Technology, 1277 Jiefang Avenue, Wuhan 430022, China; ‡ Union Hospital, Hospital, Tongji Medical College, Huazhong University of Science and Technology, Wuhan 430030, China; § Department of Rheumatology, Renji Hospital Affiliated to Shanghai Jiao Tong University School of Medicine, Shanghai 200001, China; ∥ Department of Surgery, Prince of Wales Hospital, The Chinese University of Hong Kong, Hong Kong 999077, China

**Keywords:** conductive hydrogel, infected wounds, NET, drug delivery, tissue regeneration

## Abstract

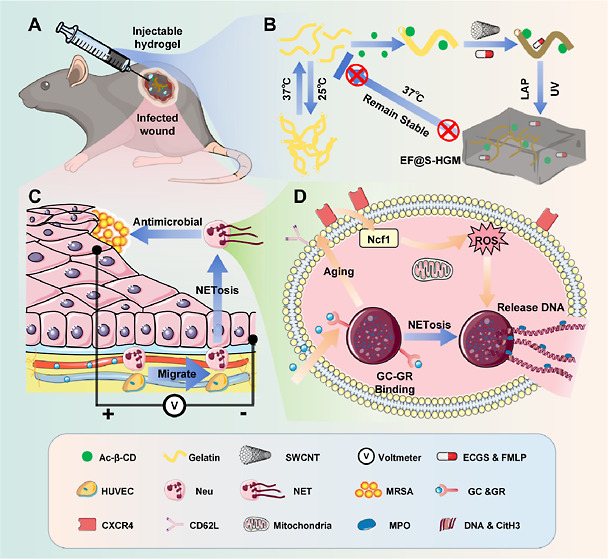

Thetreatment of infected wounds is currently a major challenge in clinical medicine, and enhancing antimicrobial and angiogenic capacity is one of the most common strategies. However, the current treatment makes it difficult to balance the antimicrobial effect in the early stage and the angiogenic effect in the later stages of wound healing, leading to an increased rate of poor prognosis. Here, we present a nanoconductive hydrogel EF@S-HGM, consisting of HGM with ECGS, FMLP, and SWCNT. The host–guest supramolecular macromolecule (HGM) hydrogel is biocompatible and can be injected in situ in the wound. The endothelial cell growth factor (ECGS) accelerates vascular remodeling and repairs wounds by promoting the proliferation of endothelial cells. N-Formyl-Met-Leu-Phe (FMLP) recruits neutrophils and increases the antimicrobial capacity. Single-walled carbon nanotubes (SWCNT) make the hydrogel conductive, enabling the hydrogel to utilize the endogenous electric field in the wound to recruit multiple kinds of cells. In addition, we found that the EF@S-HGM hydrogel activates the glucocorticoid receptor senescence pathway and promotes the formation of NET, which enhances the antimicrobial effect. As tissue-engineered skin, the conductive hydrogel EF@S-HGM is a promising material for regenerative medicine that may provide a potential option for the treatment and care of infected wounds and significantly improve patient outcomes and prognosis.

The healing process of a wound is a series of interrelated phases, including hemostasis, inflammation, proliferation, and remodeling.^1^ During the inflammation phase, various inflammatory cells, including neutrophils, accumulate, engulf microorganisms, stimulate granulation tissue and blood vessel formation, and accelerate wound repair. During the proliferation phase, endothelial cells induce vascular remodeling under the stimulation of growth factors, further speeding up the healing of wounds.^2−4^ However, when microbial toxicity is too strong, or the body’s immune function is insufficient, the inflammation phase can be prolonged, leading to delayed wound healing and failure to enter the proliferation and remodeling phases successfully.^5^ Severely infected wounds not only delay wound healing, seriously affecting the health of patients, but may also lead to pus production, redness, swelling, and severe pain, and even cause systemic symptoms and sepsis, increasing the risk of amputation.^6,7^ With the increase in antibiotic resistance, the difficulty of treating such wounds continues to rise.^8^ Therefore, a deeper understanding of the biological mechanisms and the development of effective therapeutic strategies and biomaterials have become a focus of clinical practice and scientific research to cure infected wounds.

Researchers have recently found that in addition to killing bacteria through phagocytosis and degranulation, activated neutrophils produce neutrophil extracellular traps (NETs) to phagocytose bacteria.^9,10^ In various diseases such as sepsis, autoimmunity, and cancer, excessive release of NET directly damages cells and triggers tissue inflammation. In the circulation, the formation of NET may encourage clotting, vascular obstruction, and thrombosis.^9,11^ In diabetic wounds, the massive production of NET is considered a major detriment to healing, impairing fibroblast function by activating endoplasmic reticulum (ER) stress.^12^ However, in the fight against infection and inflammation, neutrophils are one of the first immune cells to trigger an immune response. Neutrophils promote NETosis by accelerating the release of DNA fibers and granule proteins that bind and kill invading bacteria.^13,14^ Therefore, developing a strategy based on moderate activation of neutrophils may be beneficial for accelerated wound healing in infected skin wounds.

Recent studies have shown that electric fields are generated in a variety of physiological and pathological environments in the body, including wound healing, alveolar epithelial regeneration, and spinal cord injury.^15−17^ When the skin layer is broken, an endogenous electric field of about 4 μA cm^–2^ is immediately generated. Both positive charges and many repair-associated cells flow from the edge of the wound to the center, which is thought to be a wound-induced healing process.^18^ Electric fields activate multiple signaling pathways critical for wound healing, providing powerful and critically directed signals for cell migration in wound healing. The mobilization of endogenous host cells, including neutrophils and vascular endothelial cells, is critical to wound healing.^15^ Due to the lack of a stable extracellular matrix (ECM) and electric field environment in infected wounds, the infiltration of cellular components and tissue regeneration are severely hampered. Therefore, using biomaterials to mimic the tissue ECM and to create a conductive local microenvironment can solve this problem.^19^

Hydrogels are biomedical polymers with a three-dimensional molecular network structure that provide a moist environment similar to that of the ECM,^20−22^ which have led to multiple applications in drug delivery, implantation, and tissue engineering. In our previous study, we developed a host–guest supramolecular macromer (HGM) formed by physical cross-linking of acrylate-β-cyclodextrin (Ac-β-CD) and gelatin through host–guest interactions, which is a precursor form of hydrogels. The HGM shows excellent biocompatibility, cellular adaptation, and significant drug-loading capacity.^23,24^ To further optimize this effect, our team designed an EF@S-HGM hydrogel that is easy to inject and could be rapidly bonded in vitro. Its main ingredients include Ac-β-CD, gelatin, single-walled carbon nanotubes (SWCNT), N-Formyl-Met-Leu-Phe (FMLP), and endothelial cell growth supplement (ECGS). Among them, ECGS may improve the proliferation of vascular endothelial cells and coordinate matrix remodeling in the vasculature.^25^ FMLP can act as a chemokine that binds to specific receptors on the surface of neutrophils to induce neutrophil chemotaxis.^26^ The electrical conductivity of SWCNT generates microcurrents in the presence of a wound electric field, which promotes the rapid migration of multiple cells along the electrochemical gradient.^27,28^ In summary, the EF@S-HGM hydrogel combines physicoelectrical and chemopharmaceutical stimulation to modulate the inflammatory response, antimicrobial activity, and tissue regeneration. It is expected to be a comprehensive initiative for treating infected wounds, providing a viable therapeutic strategy for refractory wounds and an efficient, cost-effective, and user-friendly solution for patients.

## Results and Discussion

### Construction and Characterization of the EF@S-HGM Hydrogel

**Figure 1:**
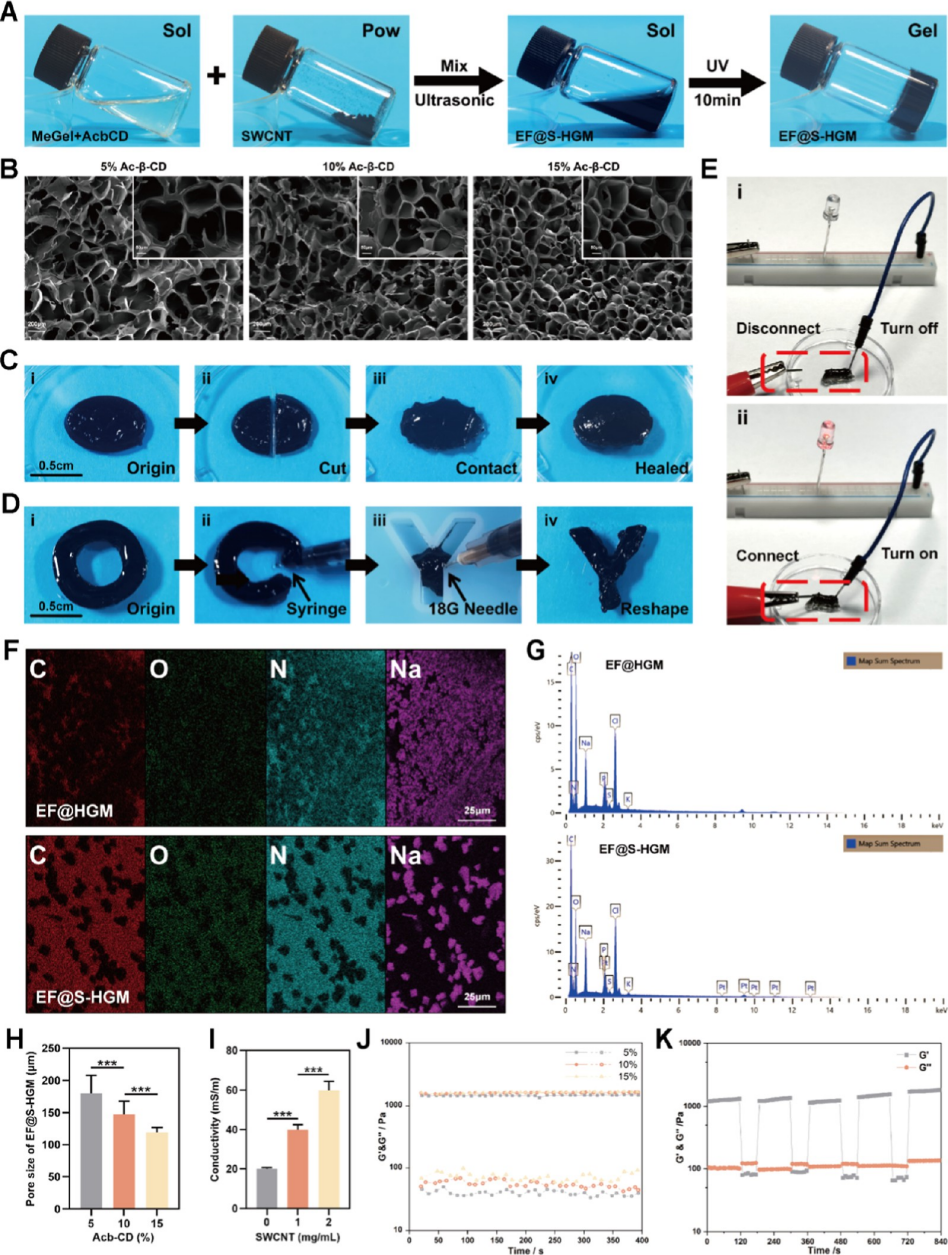
Physical characteristics of hydrogels. (A) The gelation process images of the EF@S-HGM hydrogel. (B) The SEM images of the EF@S-HGM hydrogel with fixed concentration (8%) of gel-Gelatin and different final concentrations (5%, 10%, and 15%) of Ac-β-CD. Scale bar: 200 and 50 μm. (H) The pore sizes of different concentrations of the EF@S-HGM hydrogel. (C,D) The EF@S-HGM hydrogel has good injectability and self-healing ability. Scale bar: 0.5 cm. (E,I) The EF@S-HGM hydrogel has good conductivity, which increases with the concentration of SWCNT. (F) The element mapping and (G) EDS line scan of the EF@HGM hydrogel and EF@S-HGM hydrogel. Scale bar: 25 μm. (J) The rheological time sweep of the EF@S-HGM hydrogel with different concentrations of Ac-β-CD. (K) The rheological time sweep under the cyclic low-high shear strain of the EF@S-HGM hydrogel. (gel: Gelatin-8% and Ac-β-CD: 10%). ***P < 0.001.

To synthesize the HGM hydrogel, we first solubilized gelatin polymer and Ac-β-CD in PBS buffer (pH 7.4) at 37 °C. Subsequently, the formation of the EF@S-HGM hydrogel was achieved through adding 100 μg of ECGS and 10 ng of FMLP.^29−31^ Since SWCNT is hydrophobic and not easily soluble in PBS, it is necessary to fully stir the EF@S-HGM solution to make a suspension and adjust the pH of the solution back to 7.4 with sodium hydroxide and dilute hydrochloric acid. The hydrogel suspension was then ultrasonicated in an ice bath for 10 min, which made the SWCNT uniformly dispersed and homogeneous. After centrifugation to remove air bubbles, the initiator 2-hydroxy-4′-(2-hydroxyethyl)-2 -methylpropiophenone (I2959) was added, and the mixture was transferred to the mold. Subsequently, Ac-β-CD polymerization was initiated with 365 nm UV light, forming HGM hydrogels (Figure 1A). Electron microscopy (SEM) images of EF@S-HGM hydrogels showed their unique three-dimensional porous topology (Figure 1B), and porous structures of different sizes were observed in gels containing 5%, 10%, and 15% Ac-β-CD. EF@S-HGM hydrogels containing less than 15% Ac-β-CD showed more micropores than hydrogels containing 5% Ac-β-CD. The size of these pores was large enough to allow neutrophils and human umbilical vein endothelial cells (HUVEC) to enter and shuttle through them. This structural feature was a prerequisite for subsequent experiments to load cells in the EF@S-HGM hydrogels (Figure 1H).

Our EF@S-HGM hydrogels exhibited excellent self-healing and injectable properties at 37 °C, demonstrating the stability of host–guest cross-linking (Figure 1C,D). Conventional injectable hydrogels usually require precursor solutions to be prepared in advance and injected immediately, as well as chemical cross-linking or physical interaction to form hydrogels in situ (e.g., thermal resonance hydrogels).^32^ In contrast, our EF@S-HGM hydrogels not only encapsulate the drug in them but can also be prepared in advance. It allows storage under culture conditions and can be injected into the skin as a gel at a defined time. This will provide significant benefits to physicians and patients and greatly accelerate clinical procedures. In addition, the EF@S-HGM hydrogel is able to remain in a “gel” state after passing through the narrow needle tip and conforms to the geometry at the injection site (Figure S1A). This property conforms to the tendency of human skin to deform during strain and bending, avoiding potential damage caused by diffusion or leakage of the injected material to any other locations for precise drug delivery. We also investigated the energy storage modulus (*G*′) and loss modulus (*G*″) of different hydrogels as well as the rheological time scans under cyclic low and high shear strains. These results demonstrated that the EF@S-HGM hydrogel has excellent mechanical stability (Figure 1J,K). In addition, shear viscosity and pressure strain experiments similarly illustrated the excellent rheological properties of the hydrogel, allowing it to withstand multiple compressions and stretches without damage (Figure S1B–D). This property is crucial for the integrity of the hydrogel when implanted into a traumatized area such as a weight-bearing site such as the sole of the foot or a joint.^33^

It is commonly believed that carbon nanotubes act by transmitting electrical signals and enhancing neuronal excitability. However, recent reports have shown that carbon nanotube–hydrogel composites can just rely on endogenous electric fields to recruit a wide range of cells and enhance the proliferation and differentiation of certain stem cells.^34−36^ SWCNT is a nanoconductive material consisting of monolithic graphene sheets coiled together, typically only 0.4 to 2 nm in diameter. Through the synergistic effect of van der Waals force and electrostatic force between hydrogel micropores, the number of carbon nanotubes adsorbed on the pore wall will increase, and it is not easy to fall off from the pore wall.^37^ The rapid self-healing process of the hydrogel ensures the integrity of the local electric field environment and avoids trauma to the degradation of the electrical properties of SWCNT during large deformation.^38^ SWCNT has good electron transfer properties and can effectively enhance the electrical conductivity of composites at a very low level.^39^ It improves the conductivity of hydrogels in a dose-dependent manner, which could enable EF@S-HGM hydrogels to act as wires to make a light bulb glow (Figure 1E,I and Video S1). The addition of SWNCT had no significant effect on the microstructure of the hydrogel. At the same time, elemental mapping and energy-dispersive spectroscopy (EDS) confirmed the uniform carbon distribution within the EF@S-HGM hydrogels (Figure 1F,G).

**Figure 2:**
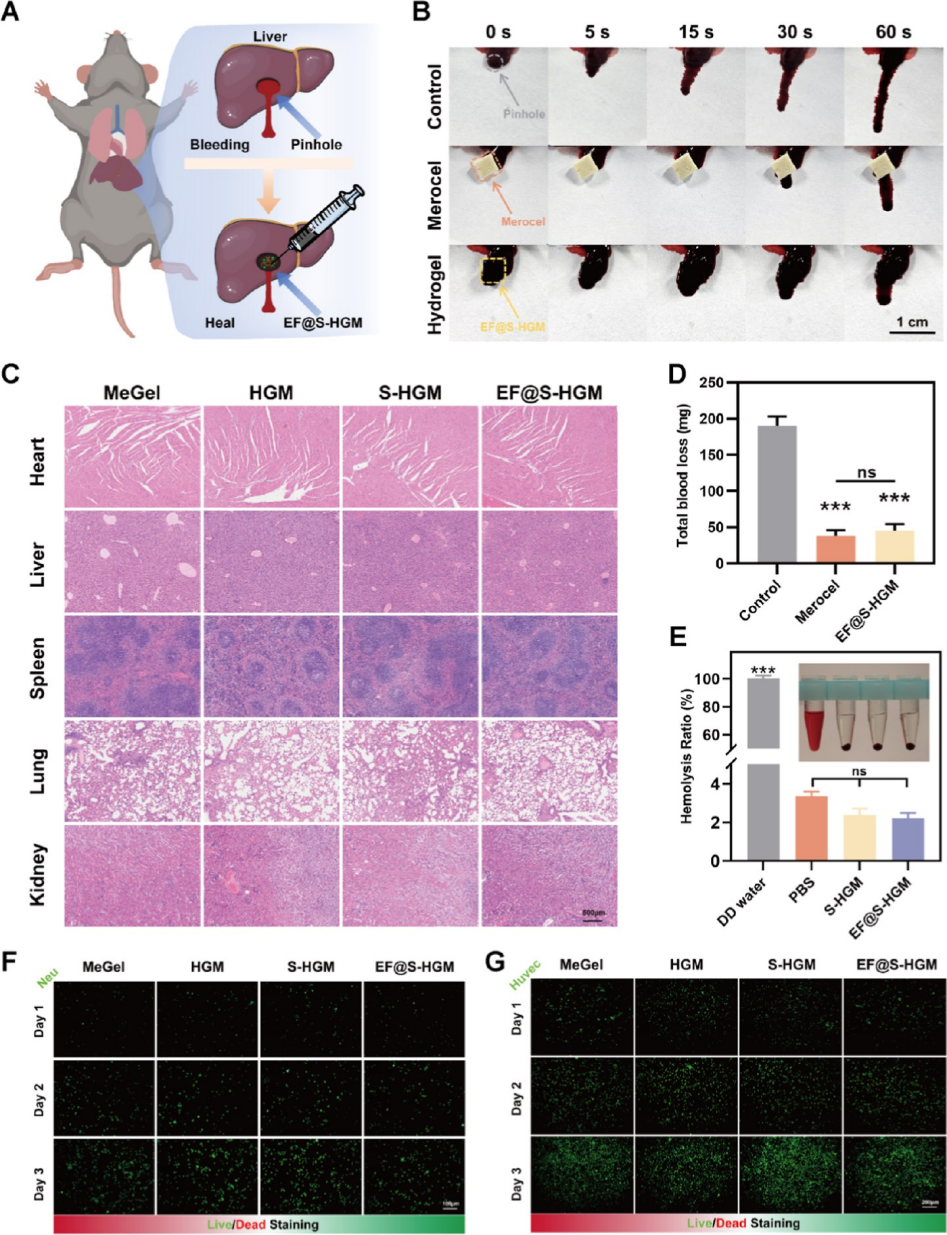
EF@S-HGM hydrogel has good biocompatibility. (A) The schematic diagram, (B) the physical image, and (D) the quantitative analysis of the mouse liver hemostasis experiment. Each group has 3 mice. Scale bar: 1 cm. (C) HE staining of the main organs of mice after injecting the hydrogel. Scale bar: 500 μm. (E) Hemolysis experiment and its quantitative analysis. (F) Live/dead staining of HL-60 and (G) HUVEC after coculturing with hydrogels for 1, 2, and 3 days. Scale bar: 100 and 200 μm. ns, nonsignificant, ***P < 0.001.

To investigate the good biocompatibility of EF@S-HGM hydrogels, we tried to validate them in vivo and in vitro.^40^ In mouse liver hemostasis experiments, we found that injection of the hydrogel at the wound site reduced blood loss, suggesting that the EF@S-HGM hydrogel could promote coagulation (Figure 2A,B,D). Also, the EF@S-HGM hydrogel did not cause hemolysis, which makes it possible not only to apply it to wounds with severe bleeding but also to use it when the wound has just happened (Figure 2E). To further investigate the toxicity to major organs, we injected methacrylated gelatin (MeGel), HGM, S-HGM, and EF@S-HGM hydrogels into different infected wounds in mice. As shown in Figure 2C, there were no significant changes in the heart, liver, spleen, lungs, and kidneys. In the early stage of infected wound healing, neutrophils are one of the first immune cells recruited to the site of inflammation, with the ability to resist bacterial proliferation and remove tissue debris.^41^ HUVEC are essential cells in the full phase of wound repair, and the amount of their vascularization greatly affects the healing familiarization.^42^ Live–dead staining experiments showed (Figure 2F,G) that human promyelocytic leukemia cells (HL-60) and HUVEC coated in EF@S-HGM hydrogels could engage with the surrounding hydrogel matrix. They also remained viable to a large extent (>95% viability).

### EF@S-HGM Hydrogel Promotes HL-60 Migration and NET Formation

**Figure 3:**
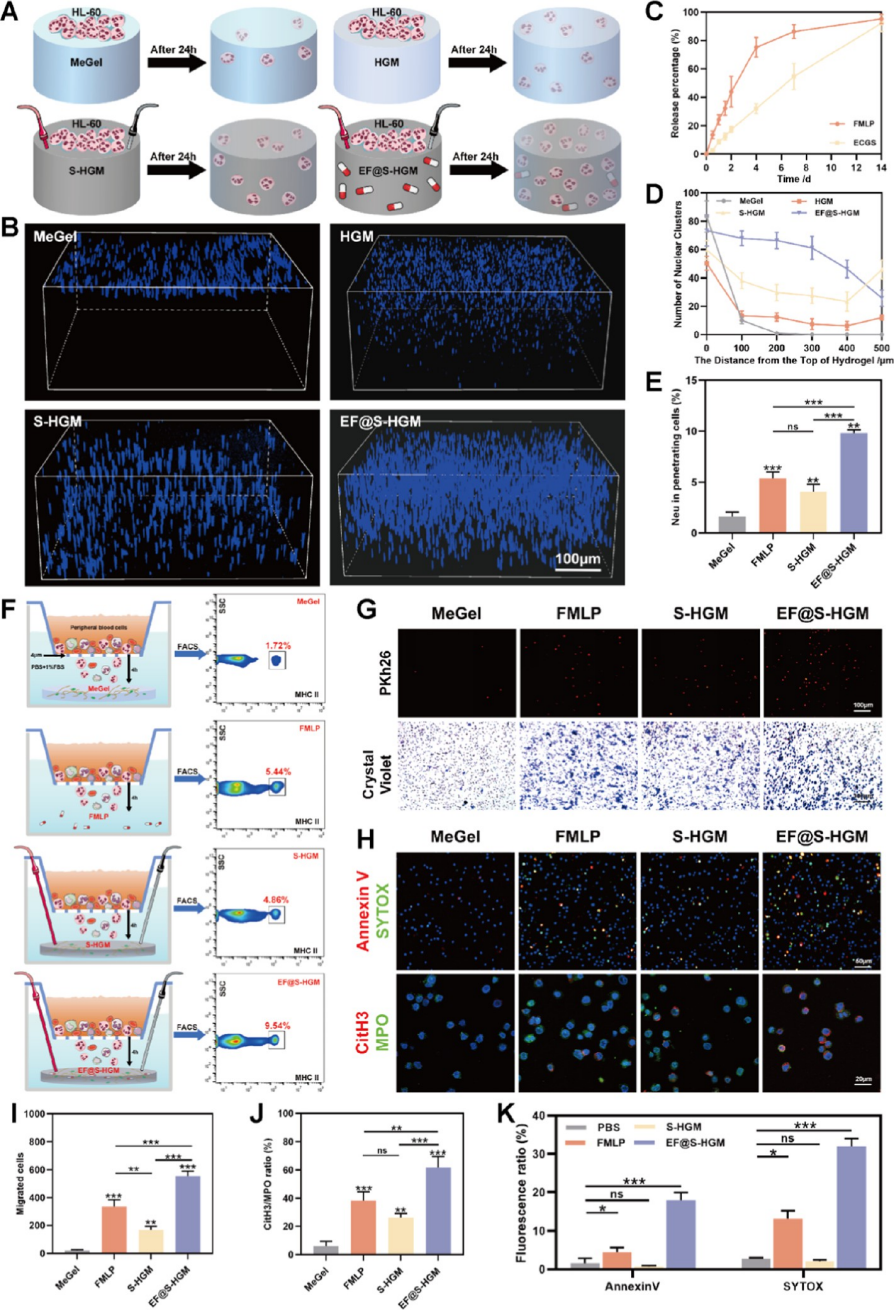
EF@S-HGM hydrogel induces the migration of neutrophils and the formation of NETs. (A) The schematic diagram of EF@S-HGM hydrogels loaded with drugs and cells. (C) Drug release curves of FMLP and ECGS. (B) Penetrating confocal 3D images and (D) distribution curve of EF@S-HGM hydrogels loaded with neutrophils. Scale bar: 100 μm. (F,E) Transwell assay and flow cytometry analysis of neutrophils in peripheral blood after coculturing with EF@S-HGM hydrogels. (G,I) PKh26 staining and crystal violet staining of neutrophils after passing through a Transwell chamber with a pore size of 4 μm. Scale bar: 100 μm. (H) Fluorescence spectra of neutrophils after cocultivating with the EF@S-HGM hydrogel, quantitative analysis of the relative proportion of (J) CitH3/MPO, and (K) quantitative analysis of the proportion of Annexin V and SYTOX double-positive cells in total cells. Scale bar: 100 and 20 μm. All results are expressed as mean ± SD, *n* = 3. ns, nonsignificant, *P < 0.05, **P < 0.01, ***P < 0.001.

Electromigration is one of the mechanisms of cell migration. Cells move in the direction of the cathode or anode depending on the cell type in response to an electric field, which is essential for embryogenesis, inflammation, wound healing, and tumor metastasis.^43^ The conventional hydrogels lack sufficient electrical conductivity, which limits signaling distances.^44^ EF@S-HGM hydrogels have reversible host–guest cross-linking properties and excellent electrical conductivity to support cell infiltration and drug release. To simulate the microelectric field of broken skin, we inserted electrodes at both ends of the hydrogel and applied a voltage of 25 mV to observe the migration infiltration ability of neutrophils in the hydrogel. As shown in Figure 3A, we designed four sets of infiltration experiments to observe HL-60 infiltration in the hydrogels. Among them, the S-HGM and EF@S-HGM hydrogel groups had electrodes inserted at both ends to simulate the endogenous microelectric field in the wound. The results (Figure 3B,D) showed that most of the HL-60 cells inoculated on the surface of the MeGel hydrogel were retained at the top of the hydrogel. The cells inoculated on HGM and S-HGM hydrogels infiltrated more downward than MeGel, indicating that the microphysical structure of HGM is more favorable for cell infiltration.^45^ Notably, cells in the S-HGM hydrogel moved along the direction of the electric field, suggesting that additional electrical stimulation had a significantly higher effect on HL-60 migration than gravitropism and free diffusion in the MeGel and HGM groups.^35^ Moreover, almost all HL-60 cells on our EF@S-HGM hydrogel infiltrated into the hydrogel, significantly higher than those of the other three groups. Under the same conditions, the cells migrated long distances within the EF@S-HGM hydrogel, suggesting that the chemokine FMLP acted on HL-60 cells, which is consistent with previous findings. Eighteen β-CD have long been used in the pharmaceutical industry.^46^ Their addition to hydrogels can improve drug solubility and bioavailability.^47^ Therefore, we tested the effect of the drug release in EF@S-HGM hydrogels. Figure 3C shows that the EF@S-HGM hydrogel can continuously release FMLP and ECGS for up to 14 days. The early release of FMLP was more favorable for the recruitment of neutrophils in the infected wounds. ECGS, at an almost constant rate, ensured the therapeutic ability of HUVEC during the full period of wound healing.

Subsequently, we assessed the ability to recruit neutrophils in the EF@S-HGM hydrogel in vitro (Figure 3E,F). Peripheral blood from mice was added to the upper chamber of Transwell containing the serum-free 1640 medium. The lower chamber was filled with PBS containing 1% FBS. Then, MeGel, FMLP, S-HGM, and EF@S-HGM hydrogel were added to the lower chamber, respectively. Electrodes were inserted, and the voltage was set to mimic the microelectric field in the trauma. The Transwell system with a pore size of 4 μm allowed free passage of peripheral blood cells. After 4 h of incubation, cells in the bottom chamber were collected for fluorescence-activated cell sorting (FACS) analysis.^48,49^ Compared to FMLP or S-HGM hydrogel groups, the neutrophil migration rate in EF@S-HGM hydrogels was significantly increased. To further verify the effect of the EF@S-HGM hydrogel on neutrophil migration, we extracted neutrophils from bone marrow alone and performed Transwell experiments immediately. After 4 h of incubation, cells were stained with Pkh26 and crystal violet, respectively. The results showed a significant increase in the migratory capacity of the cells after coculturing with the EF@S-HGM hydrogel (Figure 3G,I).

Neutrophils are the first line of defense against bacterial infections. NETosis is one of the important protective mechanisms. Early immunomodulation during infection repair is essential to fight bacteria.^50^ Neutrophils also play an important role in tissue repair. Neutrophils may release NETs upon activation by various stimuli, including cytokines secreted by multiple cells and chemokines secreted by bacteria.^51,52^ The process of NETosis mainly involves the release of large amounts of reactive oxygen species (ROS), migration of neutrophil elastase (NE) and myeloperoxidase (MPO) to the nucleus, and histone modification and uncoupling (mainly of guanylylated histone 3, CitH3).^53,54^ Based on the above properties, common detection methods for NET include the DNA stains DAPI and SYTOX and immunofluorescence staining for the marker protein CitH3 (MPO is a marker protein for neutrophils).^55,56^ Thus, we first performed SYTOX and Annexin V staining (Figure 3H,J) on neutrophils to reflect the NETosis and early apoptosis.^57,58^ Then, we performed IF staining for MPO and CitH3 in neutrophils.^59^ Co-localization analysis showed (Figure 3H,K) increased CitH3/MPO in neutrophils cocultured with EF@S-HGM hydrogels. More detailed experiments (Figure S2A–E) demonstrated that FMLP in hydrogels acts during the inflammatory phase of wound healing and affects neutrophils in a dose-dependent way. In short, these results suggest that EF@S-HGM hydrogels can promote neutrophil apoptosis and increase NET production.

### EF@S-HGM Hydrogel Induces NETosis of HL-60 Cells via the GR Senescence Pathway

For more than two decades, many researchers have been interested in the interaction of electric fields with biological cells. However, the signaling pathways involved are not yet fully understood.^44^ Under physiological conditions, glucocorticoids (GC) regulate sugar, protein, and fat metabolism, and they also regulate potassium, sodium, and water metabolism, which are essential for balancing the body’s inner environment.^60^ Researchers have found that the GC leads to rapid senescence of bone marrow adipocytes, which induces bone loss and osteoporosis, yet this process can be slowed down by inhibiting the glucocorticoid receptor (GR).^61^ In addition, excess GC modulates immunometabolism in the skeletal microenvironment, promotes the entry of GC ligand receptors into the nucleus, and inhibits bone turnover in mice.^62^ Furthermore, neutrophils exposed to stress develop a senescent phenotype characterized by high C-X-C chemokine receptor 4 (CXCR4) and low CD62L expression. And aging neutrophils produce more ROS, stimulating NETosis.^11^ Since the electric field can also be considered as a stimulus to live cells, we hypothesized that the promotion of NETosis by the EF@S-HGM hydrogel might be related to GR, CXCR4, CD62L, and other related stress and aging processes.^63^

**Figure 4:**
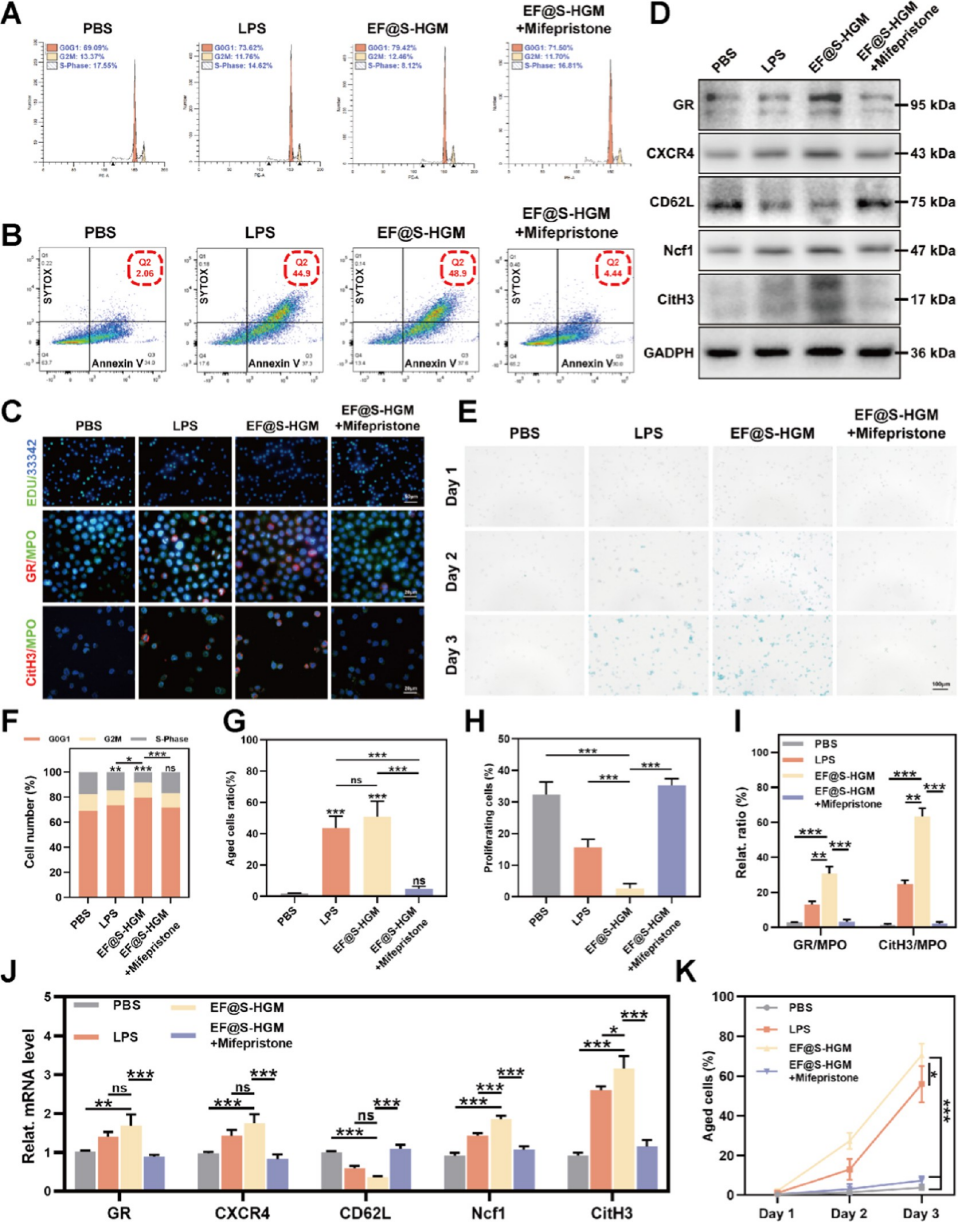
EF@S-HGM hydrogel promotes NETosis through the GR aging pathway. (A,F) Periodic flowchart of HL-60 after coculturing with the EF@S-HGM hydrogel. (B,G) HL-60 apoptotic flow cytometry and quantitative analysis. (C,I) Different fluorescence staining patterns of Neu (EDU, GR/MPO, CitH3/MPO). Scale bar: 50 μm, 20 μm, 20 μm. (H) Quantitative analysis of the proportion of proliferating cells in EDU. (D) Western blot of GR channel-related proteins and (J) qPCR detection results of related mRNA. (E) β-galactosidase staining of Neu and (K) the proportion of aged cells. Scale bar: 100 μm. All results are expressed as mean ± SD, *n* = 3. ns, nonsignificant; *P < 0.05; **P < 0.01; ***P < 0.001.

Lipopolysaccharide (LPS) is a known bacterial pathogen that activates the innate immune response, binds to neutrophils, and increases apoptosis. Mifepristone is a common GC antagonist that modulates the GR signaling pathway.^64,65^ We cocultured HL-60 cells with PBS, LPS (100 ng mL^–1^), EF@S-HGM hydrogels, and EF@S-HGM hydrogels with mifepristone, respectively. The proliferation capacity of HL-60 cells was first analyzed. The cell cycle detection by flow cytometry showed a significant reduction in the transformation of HL-60 cells to G2 and S phases 24 h after EF@S-HGM hydrogel treatment (Figure 4A,F), and EdU-positive nuclei were also significantly reduced (Figure 4C,H). This result suggests that the proliferation capacity of HL-60 cells was inhibited in the EF@S-HGM hydrogel. However, this inhibitory effect could be largely reversed by mifepristone to the level of the PBS group. We then examined the apoptosis and senescence of HL-60 cells. The proportion of cells that were double positive for SYTOX and Annexin V was significantly higher after incubating HL-60 cells with the EF@S-HGM hydrogel for 24 h compared to the PBS group. However, adding mifepristone returned the proportion of cells to the level of the PBS group (Figure 4B,G). The results of β-galactosidase staining also showed that the proportion of senescent cells in the EF@S-HGM hydrogel was as high as 68.3%. However, only 4.36% and 6.29% were found in the PBS and EF@S-HGM hydrogel with mifepristone groups (Figure 4E,K). Subsequently, Western blot (WB) and qRT-PCR demonstrated how the EF@S-HGM hydrogel prompted NETosis (Figure 4D,J). EF@S-HGM hydrogel treatment increased the expression of GR, CXCR4, Ncf1, and CitH3 proteins in HL-60 cells but decreased the expression of CD62L protein. Finally, immunofluorescence colocalization analysis also illustrated that mifepristone alleviated the increase in GR/MPO and CitH3/MPO in neutrophils caused by EF@S-HGM hydrogel coculture. Taken together, these results suggest that the conductive hydrogel EF@S-HGM promotes NETosis through the GR senescence pathway (Figure 4C,I).

### Effective Antimicrobial Capacity of the EF@S-HGM Hydrogel

**Figure 5:**
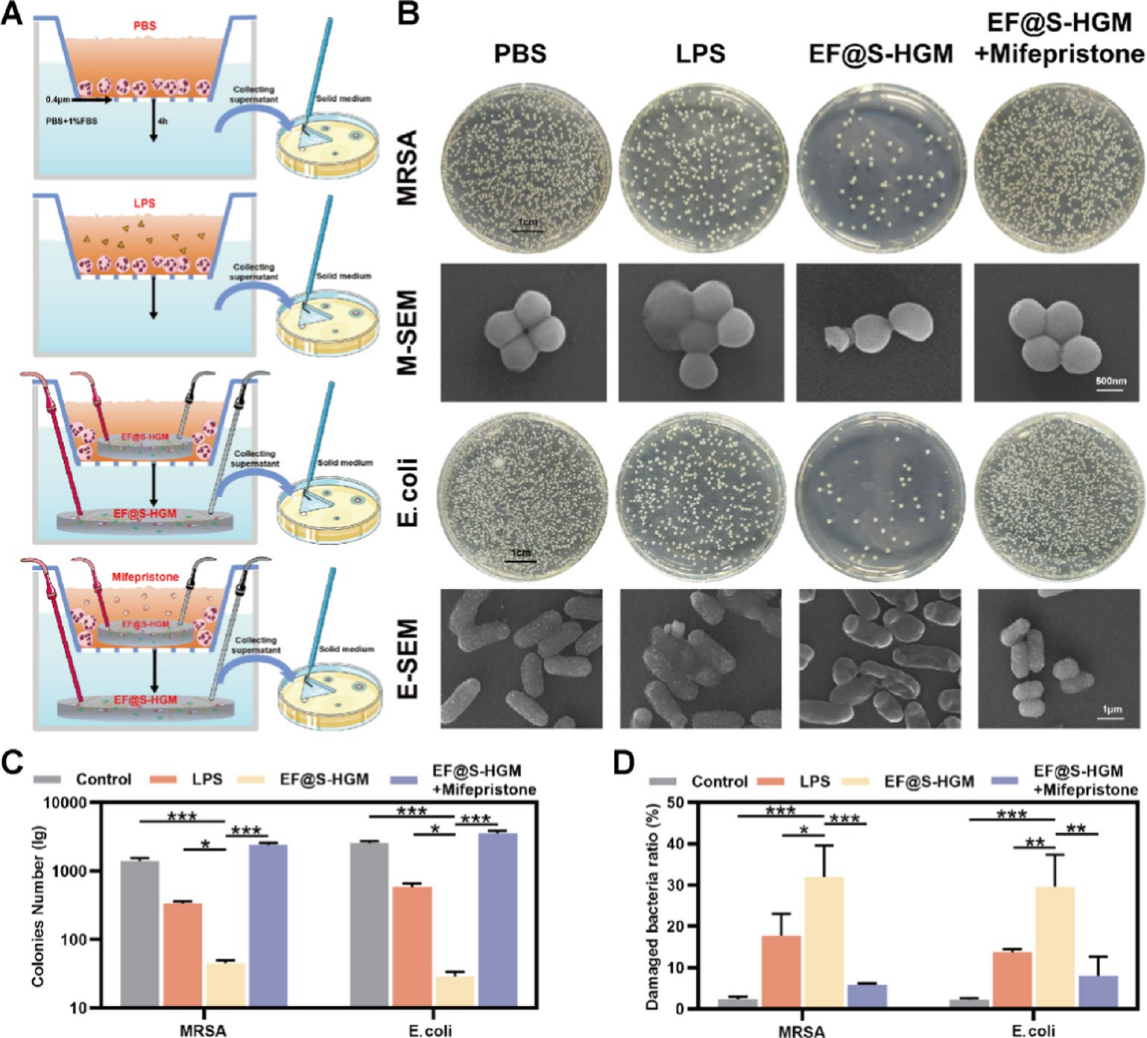
EF@S-HGM hydrogel has good antibacterial ability. (A) The schematic illustration of the coculture system and the bacterial coating with a cocultured solution. (B) Physical and electron microscopy images of MRSA and *E. coli* cultured for 12 h. Scale bar: 1 cm, 500 nm, 1 μm. (C,D) Quantitative analysis of bacterial count and the proportion of structurally damaged bacteria. All results are expressed as mean ± SD, *n* = 3. *P < 0.05, **P < 0.01, ***P < 0.001.

To assess the antimicrobial capacity of the EF@S-HGM hydrogel in vitro (Figure 5A), we added HL-60 cells with a serum-free 1640 medium to the upper chamber of the Transwell and then added PBS, LPS, EF@S-HGM hydrogel, and EF@S-HGM hydrogel with mifepristone to the upper chamber of the Transwell system, respectively. The lower chamber was filled with PBS solution containing 1% FBS. Electrodes were inserted at both ends of the hydrogel, and the voltage was set at 25 mV to simulate the microelectric field in the trauma. A Transwell system with a pore size of 0.4 μm prevents neu from passing through but allows NETs to migrate freely into the lower chamber.^14^ To counteract the chemotactic effect of the EF@S-HGM hydrogel on NETs in the upper chamber, we also added the EF@S-HGM hydrogel in the lower chamber. After 4 h of migration, the lower chamber solution containing NET was cocultured with bacteria to test its antimicrobial capacity. The results showed that the EF@S-HGM hydrogel group exhibited excellent antimicrobial capacity, with 96.7% and 98.8% inhibition rates against methicillin-resistant *Staphylococcus aureus* (MRSA) and *Escherichia coli*, respectively (Figure 5B,C).

The morphology of *S. aureus* and *E. coli* under different treatments was observed using SEM. As shown in Figure 5B,D, compared with the use of PBS and LPS, the HL-60 supernatant treated with the EF@S-HGM hydrogel severely damaged the bacterial cell wall against both *S. aureus* and *E. coli*, and this disruptive effect was largely mitigated by the addition of mifepristone.

### EF@S-HGM Hydrogel Promotes HUVEC Repair

**Figure 6:**
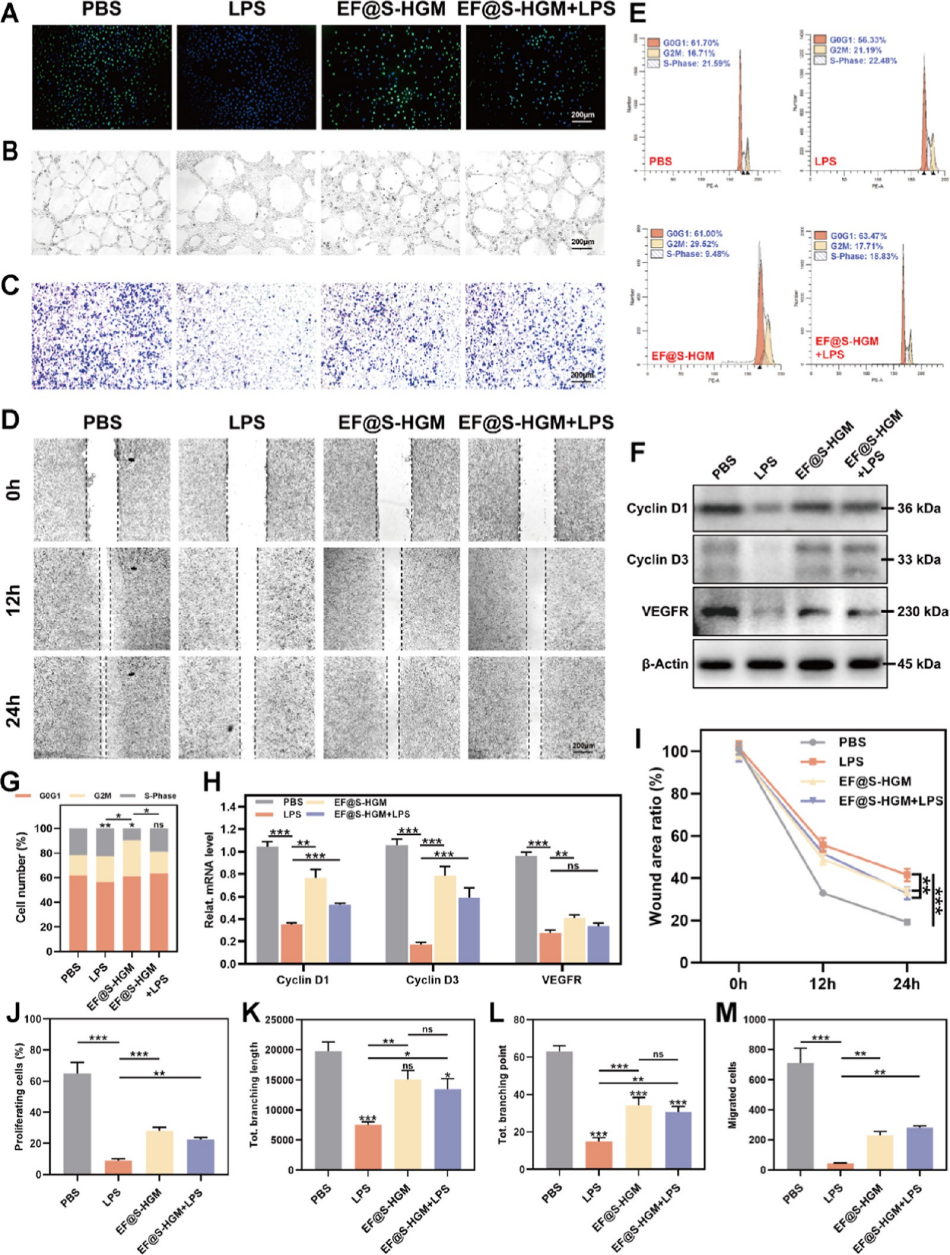
EF@S-HGM hydrogel promotes HUVEC repair. (A) EdU staining images and (J) quantitative analysis of HUVEC after cocultivating with the EF@S-HGM hydrogel. Scale bar: 200 μm. (B) Tube formation test of HUVEC after cocultivation and (K,L) quantitative analysis. Scale bar: 200 μm. (C) Transwell crystal violet image of HUVEC after cocultivation and (M) quantitative analysis. Scale bar: 200 μm. (D) Wound healing test images of HUVEC after cocultivation and (I) quantitative analysis. (E) Periodic flowchart of HUVEC after cocultivation and (G) quantitative analysis. (F) WB band plots of related protein of HUVEC after cocultivation and (H) qPCR detection results of related mRNA. Scale bar: 200 μm. All results are expressed as mean ± SD, *n* = 3. ns, nonsignificant, *P < 0.05; **P < 0.01; ***P < 0.001.

When a wound in the inflammation phase successfully controls infection, it enters the proliferation phase. At that time, the local blood supply of the wound is a determinant of wound healing.^66^ A large-area trauma experiment in mice demonstrated that electrical stimulation therapy has emerged as an effective strategy to promote wound healing by enhancing the cellular electrosensitivity. This therapy plays an important role in regulating proliferation, migration, and angiogenic differentiation.^67^ Another porcine-based experiment also confirmed that by remodeling the electric field at the site of trauma, conductive dressings can induce a strong chemotactic migratory response in epidermal cells, accelerating reepithelialization of the trauma and helping to improve the quality of healing of new skin during the remodeling phase.^68^ To investigate whether the EF@S-HGM hydrogel has a promotional effect on HUVEC proliferation, migration, and angiogenesis in the presence of bacterial infection, we cultured HUVEC under LPS conditions. First, we analyzed the proliferation capacity of HUVEC. We observed that EdU-positive nuclei were significantly reduced when treated with LPS and that this effect of LPS could be largely reversed (Figure 6A,J). Interestingly, the results of the angiogenesis assay in vitro also showed that the tube structure was improved with the addition of the EF@S-HGM hydrogel, with an increased number of tubes formed and a prolonged tube length compared with the LPS group (Figure 6B,K,L). Next, we further verified the effect of the EF@S-HGM hydrogel on cell migration. We performed Transwell experiments, as shown in Figure 3F. After 24 h of incubation, the experimental results demonstrated that cell migration was obviously enhanced by adding the EF@S-HGM hydrogel (Figure 6C,M). We performed scratch experiments on HUVEC to evaluate the in vitro wound closure effect of the EF@S-HGM hydrogel. After 24 h of incubation, wound closure was slow in the LPS group, whereas faster wound closure was observed in the EF@S-HGM hydrogel group under LPS conditions (Figure 6D,I). In addition, cell cycle assays by flow cytometry indicated a dramatic increase in the transformation of HUVEC toward G2 and S phases after EF@S-HGM hydrogel treatment (Figure 6E,G). Finally, Western blotting and qRT-PCR results showed that LPS treatment decreased the expression of CyclinD1 and CyclinD3 proteins in HUVEC. This trend could be partly counteracted by EF@S-HGM hydrogel treatment (Figure 6F,H). More detailed experiments showed that ECGS in hydrogels exerted importance in the repair phase of wound healing and affected HUVEC cells in a dose-dependent manner (Figure S3A–H). In conclusion, the EF@S-HGM hydrogel attenuates the deleterious effects of LPS and promotes cell proliferation, migration, and angiogenesis, thereby accelerating wound healing.

### Significant Therapeutic Effect of the EF@S-HGM Hydrogel on Infected Wounds in Vivo

**Figure 7:**
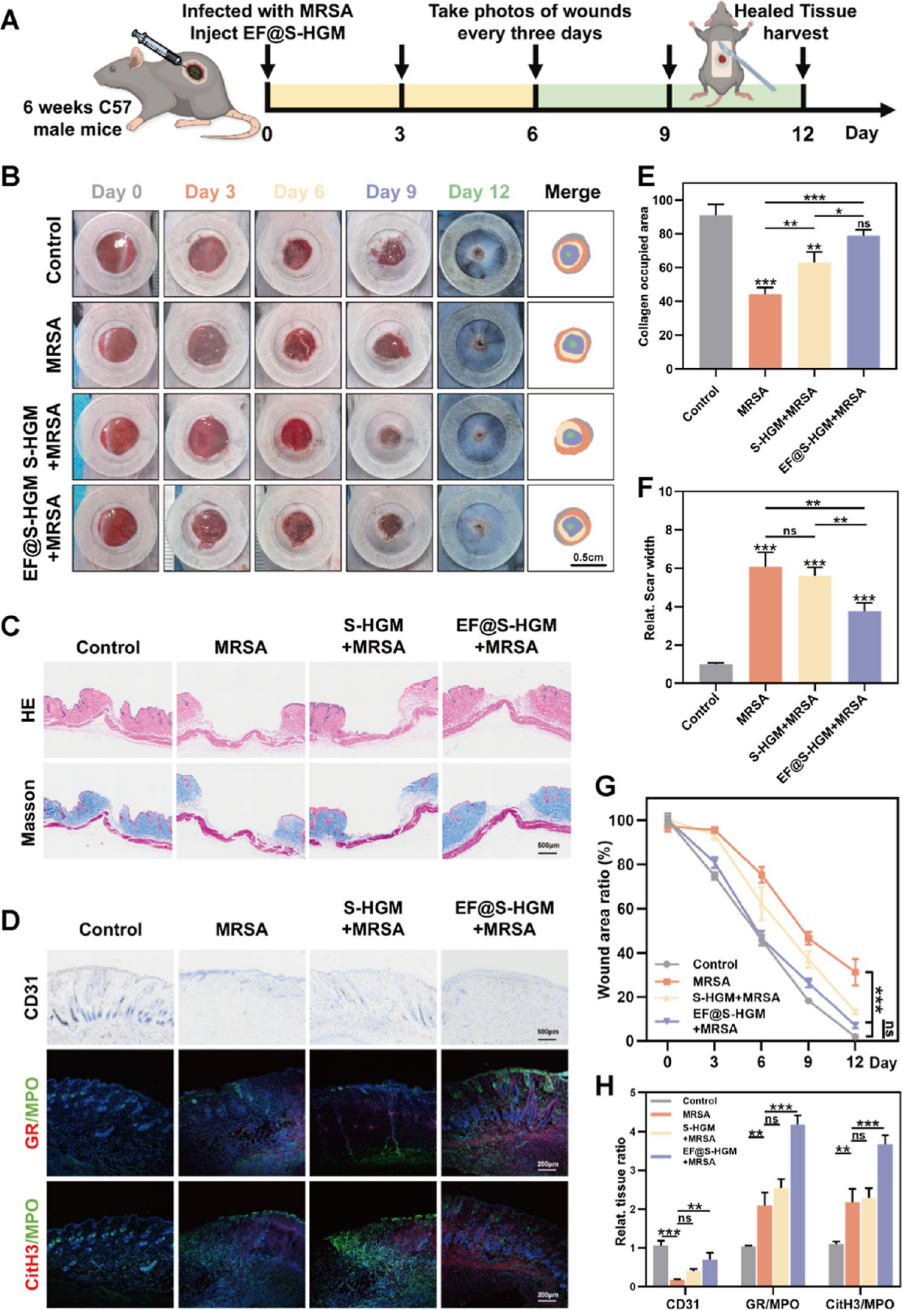
EF@S-HGM hydrogel can promote the healing of infectious wounds *in vivo*. (A) The schematic illustration of the infectious wound model and the injection of the EF@S-HGM hydrogel. (B,G) Representative general images and the statistical results of the wound closure. Scale bar: 0.5 cm. (C) Representative images of HE and Masson staining of infectious wounds, (E) quantitative analysis of collagen occupied area, and (F) quantitative analysis of scar width in each group. Scale bar: 500 μm. (D) CD31 immunohistochemistry, GR/MPO and CitH3/MPO immunofluorescence images, and (H) quantitative analysis of infectious wounds. Each group has 3 mice. Scale bar: 500 μm, 200 μm, 200 μm. All results are expressed as mean ± SD, *n* = 3. ns, nonsignificant. *P < 0.05; **P < 0.01; ***P < 0.001.

After verifying in vitro that the EF@S-HGM hydrogel has significant antimicrobial activity and pro-restorative effects, we established a mouse model of infected wounds to test whether the healing of infected wounds could be accelerated in vivo (Figure 7A). After each C57/6J mouse was dorsally depilated, the entire skin layer was excised along a 1 cm diameter circle in the center of the back properly. The wound area was photographed and measured on days 0, 3, 6, 9, and 12 (Figure 7B,G). Whole-layer wounds on the backs of mice were dressed with the MeGel hydrogel loaded with PBS (control group), MeGel hydrogel loaded with MRSA (MRSA group), S-HGM hydrogel loaded with MRSA (S-HGM + MRSA group), and EF@S-HGM hydrogel loaded with MRSA (EF@S-HGM + MRSA group). No animals died or showed signs of abnormality after day 0 of surgery. During the inflammation phase (days 3 and 6), infected mice in the MRSA group had more pronounced bacterial scabs after wounding than in the other groups, indicating that the presence of bacteria significantly prolonged the inflammatory phase of wound healing. During the repair phase (days 9 and 12), the wounds of mice in the MRSA group exhibited a slower healing rate, whereas treatment with the EF@S-HGM hydrogel significantly accelerated the wound healing process. More detailed experiments demonstrated the period of the respective actions of FMLP and ECGS in the hydrogels, similar to the results of previous in vivo experiments (Figure S4A,B). 12 days after injury, besides direct monitoring of the wound area, various histological experiments were performed to explore the features of the wound tissue. Hematoxylin–eosin (HE) and Masson staining images of wounds processed using the above methods are displayed in Figure 7C. Among the mice with infected wounds on day 12 postinjury, the narrowest scar width was observed in the EF@S-HGM + MRSA group, which almost reached the level of the control group, followed by the S-HGM + MRSA group (Figure 7F). The effect on collagen deposition and remodeling was examined by Masson staining, especially in the EF@S-HGM + MRSA group, which showed enhanced and regularly arranged collagen deposition on day 12. In contrast, wounds in the MRSA group were infiltrated with inflammatory cells and displayed a disorganized appearance (Figure 7E).

Forming new blood vessels is crucial for transporting nutrient factors to the wound site during wound healing. Therefore, we next investigated whether the EF@S-HGM hydrogel treatment could promote angiogenesis at the wound site. As shown in Figure 7D, more CD31-labeled blood vessels were observed in the EF@S-HGM + MRSA group compared to the MRSA group. In addition, the expression levels of GR, CitH3, and MPO in wound tissues were analyzed (Figure 7D,H). To explore the effect of the EF@S-HGM hydrogel on neutrophils in vivo, immunofluorescence for GR, CitH3, and MPO was performed. Consistent with the in vitro experiments, NETs were detected in the EF@S-HGM group, with a significant difference from that of the MRSA group. These findings suggest that the EF@S-HGM hydrogel helps heal infected wounds through various mechanisms such as collagen deposition, angiogenesis, and NETosis.

## Conclusions

Infected wounds are one of the great challenges in clinical management, mainly because they are susceptible to bacterial attack and are often drug-resistant, which exacerbates the infection and delays wound healing, increasing the cost of care and the risk of patient death. Therefore, properly caring for wounds and using beneficial dressings are particularly important for the treatment. In this study, we developed a nanoconductive hydrogel, EF@S-HGM, to treat infected wounds. This hydrogel is electrically conductive and can accelerate wound healing using biomicroelectric fields. By releasing the chemokine FMLP, the hydrogel effectively recruits neutrophils and enhances NETosis to fight against microbial pathogens. Meanwhile, the hydrogel promotes endothelial cell proliferation and angiogenesis to improve the healing of infected wound healing. Furthermore, we validated the excellent biocompatibility and bacteriostatic ability of the EF@S-HGM hydrogel. We also explored how the EF@S-HGM hydrogel induces NETosis—to activate the GR senescence pathway to promote neutrophil apoptosis. The easy drug injection in situ and excellent mechanical properties also facilitate clinical applications in future translations. Overall, the EF@S-HGM hydrogel provides a comprehensive strategy for the clinical management of infected wounds and is expected to advance the widespread use of conductive biomaterials in wound care and regenerative medicine.

## Experimental Section

### Materials

#### Aladdin

β-Cyclodextrin (β-CD), acrylate chloride, and hydrogen peroxide (H_2_O_2_) were obtained.

#### Sigma

Gelatin (type A, from porcine skin, isoelectric point: 7 × 10^9^, Cat. no.: G1890–500G), methacrylic anhydride, dimethylmalenic acid (DMMA), deuterium oxide (D2O), dimethyl sulfoxide-d6 (DMSO-*d*_6_), dexamethasone (Dex), 2-hydroxy-4′-(2-hydroxyethoxy)-2-methylpropiophenone (I2959), 40,6-diamidino-2-phenylindole (DAPI), 3-(trimethoxysilyl)propyl methacrylate, nitrocellulose, paraformaldehyde (PFA), Triton X-100, sodium thiosulfate, triethyl amine (TEA), ethidium bromide, hyaluronidase, Percoll (P4937), endothelial cell growth supplement (ECGS), and N-formyl-Met-Leu-Phe (FMLP) were obtained.

#### Jenkem

Poly(ethylene glycol) diacrylate (PEGDA) was obtained.

#### Gibco

Phosphate-buffered saline (DPBS), a-minimum essential medium (DMEM), penicillin, streptomycin, l-glutamine, calcineurin, fetal bovine serum (FBS, #10099141), and TRIzol were obtained.

#### Thermo Fisher Scientific

RPMI 1640, 24-well Transwell system, BCA protein assay kit, calcium colorimetric assay kit, revertAid First strand cDNA synthesis kit, dimethylformamide (DMF), dimethyl sulfoxide, acetone, hydrochloric acid (HCl), sodium hydroxide (NaOH), mouse anti-CitH3, rabbit anti-MPO, goat anti-GR, and FITC-labeled Cy3 secondary antibody were obtained.

#### Vector Lab

Peroxidase substrate kit DAB and vectastain ABC kit were obtained.

#### Beyotime

A calcium Xanthophyll/PI Cell Viability/Cytotoxicity Assay Kit, SDS-PAGE Gel Preparation Kit, Hoechst 33342 (C1025), and Matrigel (C0372) were obtained.

#### Biotronik

An Annexin V-mCherry/SYTOX Green Kit (C1070S) and a Cell Cycle and Apoptosis Detection Kit (C1052) were obtained.

#### Aspen

Protease, phosphatase inhibitors, and horseradish peroxidase (HRP) were obtained.

#### Millipore

NC membranes were obtained.

#### Takara

A Takara RT Master Mix Kit and a 2x SYBR Green qPCR Mix Kit were obtained.

#### Ribobio

A 5-Ethyl-2′-deoxyuridine (EdU) doping assay kit (C10310) was obtained.

#### Abclonal

Antibodies to GR (A19583), MPO (A1374), CitH3 (A17562), CXCR4 (A19035), CD62L (A1622), Ncf1 (A5143), GADPH (A19056), Cyclin D1 (A1301), Cyclin D3 (A3989), VEGFR (A1277), and β-actin (AC038) were obtained.

#### Abcam

Antibodies anti-CD31 (ab28364) were obtained.

### Preparation of the MeGel Hydrogel

8% methacrylated gelatin and 0.05% 2-hydroxy-4′-(2-hydroxyethoxy)-2-methylpropiophenone (I2959) were added to PBS at 37 °C. After complete dissolution, the gel was transferred to a polyvinyl chloride mold and cooled to 25 °C. Then, the MeGel gel was irradiated with 365 nm ultraviolet (UV) light for 10 min (10 mW cm^–2^).

### Synthesis of Acrylic Acid b-Cyclodextrin (Ac-β-CD)

Ten g of β-cyclodextrin (β-CD) was added to 150 mL of DMF, followed by 7 mL of TEA. The mixture was stirred and cooled to 0 °C, then 5 mL of acrylic acid was added to the solution. After being stirred for 12 h, the mixture was filtered to remove trimethylamine hydrochloride. A clear solution was obtained by rotary evaporation under vacuum, and then the clear solution obtained was concentrated to about 20 mL. After that, the solution was added into 600 mL of acetone to precipitate the modified cyclodextrin. The residue was washed several times with acetone and dried under vacuum for 3 days to obtain Ac-β-CD.

### Preparation of the HGM Hydrogel

According to the experimental requirements, different proportions of gelatin and Ac-β-CD were dissolved in PBS at 37 °C to obtain a mixed solution of fixed concentration (8%) gelatin and different concentrations (5%, 10%, and 15%) of Ac-β-CD. Then, 100 μg of ECGS and 10 ng of FMLP were added to the mixed solution, which was fully shaken at 37 °C for 10 min until it was completely dissolved. This was named the EF@HGM hydrogel solution. Different doses (0, 1, 2 mg mL^–1^) of single-walled carbon nanotubes (SWCNT) (0.8–1.2 nm in diameter; 100–1000 nm in length, purchased from Engineering For Life) were doped into the EF@HGM hydrogel solution. Due to the hydrophobicity of SWCNT, it is difficult to disperse it with water. Therefore, to dissolve SWCNT completely, first, the EF@S-HGM hydrogel solution was thoroughly mixed by stirring and the pH of the solution was adjusted to 7.4 with sodium hydroxide and dilute hydrochloric acid. Then, the EF@S-HGM hydrogel solution was ultrasonicated in an ice bath for 10 min so that the SWCNT was homogeneously dispersed. Centrifugation was carried out at 600 rpm for 5 min to remove the air bubbles. 0.05% of initiator I2959 was added. The mixture was transferred into a PVC mold at 37 °C, cooled to 25 °C, then irradiated with 365 nm ultraviolet (UV) light for 10 min (10 mW cm^–2^), and named EF@S-HGM hydrogel.

### Rheological Test of the Hydrogel

The rheological properties of hydrogels were measured by using a rheometer (Haake Mars40, Germany). Frequency scans (0.1–10 Hz) were carried out on different groups of hydrogels. In the shear viscosity test, the samples were subjected to four cycles of continuous shear at 0.1% strain (120 s) and 1000% (1060 s), from which the energy storage modulus (*G*′) and loss modulus (*G*″) were obtained to calculate the rheological properties of the hydrogels.

### Physical Characterization

To get the microstructure of the hydrogel, we first made the hydrogel samples into columns, freeze-dried them using a lyophilizer, and then froze them in liquid nitrogen for cross-section preparation. The samples were tightly sealed to prevent condensation from collapsing the structure and stored temporarily at −20 °C. The samples were then frozen in liquid nitrogen for cross-section preparation. Microstructural images of the hydrogels were obtained by using SEM (ZEISS GeminiSEM 300, Germany). Tests such as elemental mapping and ED line scanning were performed.

### Conductivity Studies

The electronic conduction element is represented by pure resistance (Re). We treat the ionic and electronic conductivities as independent, leading to a parallel arrangement of Re and the ionic conductivity. We determine the conductivity of a material by the relationship between its dimensions and its electrical conductivity
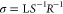
where *L* represents the electrode distance, *S* is the cross-sectional area of the material, and *R* is the resistance.

### Animals and Wound Healing and Repair Assessment In Vivo

Six-week-old male C57BL/6J mice were purchased from the Laboratory Animal Center of Tongji Medical College, Huazhong University of Science and Technology, China. They were housed under specific pathogen-free (SPF) conditions. All animal experiments were approved by the Institutional Animal Care and Use Committee (IACUC) of the Tongji Medical College. The mice were randomly divided into 4 groups, and different wound operations were taken. They were photographed and measured with calipers on the zeroth, third, sixth, ninth, and 12th days after wound establishment, respectively. ImageJ was used to calculate the percentage of the wound area
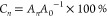


*C*_*n*_ is the proportion of wound area, *A*_*n*_ is the wound area on day n, and *A*_0_ is the wound area on day 0.

### Hemostatic Assay

Six-week-old male C57BL/6J mice were tested for the hemostatic ability of the EF@S-HGM hydrogel. After the mice were anesthetized with 10% chloral hydrate, an incision was made in the abdomen of the mice to expose the liver. The plasma around the liver was carefully removed to avoid blood volume measurement errors. A filter paper of predetermined weight (*W*_0_) was inserted under the liver, and an 18 g needle was used to puncture the liver for bleeding. The EF@S-HGM hydrogel was then injected into the wound. The control group was left untreated, and the hemostatic sponge merocal group served as a positive control. The liver wounds were photographed at 0, 5, 15, 30, and 60 s, and the blood-accumulating filter paper (*W*_1_) was weighed after 60 s. Blood loss was calculated as the weight gain of the filter paper (*W*_1_ – *w*_0_).

### Hemolysis Assay

To verify the biocompatibility of S-HGM and EF@S-HGM hydrogels, the peripheral blood of mice was stirred with a glass rod to remove fibrinogen to make defibrinated blood. A 0.9% sodium chloride solution was added to wash the erythrocytes about 2–3 times to make a 4% suspension. ddH_2_O, PBS, the S-HGM hydrogel, and the EF@S-HGM hydrogel were added, respectively. The mixture was incubated in a thermostat for 1 h. The incubation conditions were set to 37 °C and 60 rpm. The hemolysis phenomenon was observed.

### Histological Analysis

After injecting PBS or the EF@S-HGM hydrogel into 6 week-old male C57BL/6J mice for 14 days, the main organs of mice, heart, liver, spleen, lungs, and kidneys were taken for histological processing. Sections were stained with hematoxylin–eosin (HE). On day 12, wound tissue samples collected from mice were taken, fixed in 4% PFA, and embedded in paraffin. Sections (5 μm) were stained with hematoxylin and eosin (H&E) and Masson stain. Imaging was performed under an EPSON V600 digital scanner.

### Cell Culture

C57BL/6 mice were provided by the Animal Experiment Center of Tongji Medical College, Huazhong University of Science and Technology. Femoral specimens were taken, and a syringe flushed the bone marrow with a cell culture solution. The erythrocytes were lysed, and the bone marrow (BM) cells were isolated by centrifugation (400 g, 30 min) using a Percoll gradient (63% and 85%). The cells in the middle layer were collected and washed for >90% purity neutrophils.

HL-60 cells and HUVEC were donated by the Translational Research Laboratory of the Union Hospital of Huazhong University of Science and Technology, China. They were cultured in whole medium RPMI 1640 containing 10% FBS and placed in a humidified chamber grown at 37 °C with 5% CO_2_.

### 3D Reconstruction of the Hydrogel

10 μL of 1 × 10^9^ mL^–1^ of HL-60 cells was added to 1 mL of the hydrogel and irradiated with 365 nm UV light (10 mW cm^–2^, 5 min) to gelatinize it. A complete medium (containing 10% FBS, 1% penicillin/streptomycin, and 1640 medium) was used for 3D in vitro culture. During the in vitro culture process, the 3D cell culture samples were stained for nuclei with Hoechst 33342 and finally scanned and reconstructed with a confocal microscope for the 3D images.

### Cell Viability

HL-60 cells were inoculated in 96-well plates at 5 × 10^3^ cells per well. After 24 h, cells were cocultured with different hydrogels. Cell viability was tested by the Calcium Xanthophyll/PI Cell Viability/Cytotoxicity Assay Kit. After the cells were incubated for 30 min, live or dead cells were observed under a fluorescence microscope.

### Transwell Migration Assay

A 24-well Transwell system containing a 4 μm pore size filter was used to assess the migration capacity. Briefly, HL-60 cells with the serum-free medium were inoculated in the upper chamber. The medium containing drugs or the EF@S-HGM hydrogel was added as a nutrient elicitor in the lower chamber. After incubation, cells in the lower chamber were removed for flow cytometry. Filters were fixed with 4% PFA at room temperature. The migrated cells were stained with PKh26 and crystal violet and observed under a fluorescence microscope (Olympus, Japan).

### FCM Examination

We used the Annexin V-mCherry/SYTOX Green kit and the Cell Cycle and Apoptosis Detection Kit. First, following the manufacturer’s instructions, cell samples were washed twice with PBS immediately after collection, followed by fixation with 1% bovine serum albumin (BSA) for 30 min to block nonspecific binding. Subsequently, the cell samples were incubated for 20 min in the dark with the fluorescent labeling antibody provided in the Beyotime kit to label the target proteins. After incubation, the cells were washed with PBS to remove unbound antibodies. Finally, the samples were analyzed using a flow cytometer and Flowjo instrument to obtain data on the expression of cell surface markers. All experiments were repeated three times to ensure reproducible and accurate results.

### Immunofluorescence

For immunofluorescence staining, cells cultured on slides were first fixed with 4% *cis*-dialdehyde for 20 min. After that, the cells were treated with PBS solution containing 0.1% Triton X-100 for 10 min to permeate the cell membrane. After the nonspecific sites were sealed with 3% BSA for 30 min, the cells were stained with the following primary antibodies: mouse anti-CitH3 (1:5000), rabbit anti-MPO (1:2000), and goat anti-GR (1:2000). The cells were incubated with primary antibody staining at 4 °C overnight. The next day, the cells were incubated with FITC-labeled Cy3 (1:2000) secondary antibody for 1 h. Finally, nuclei were labeled with DAPI. Images were observed and captured by fluorescence microscopy, and data were analyzed by using Flowjo.

### Western Blot

Cell samples were lysed with a buffer containing a 1% mixture of protease and phosphatase inhibitors. The SDS-page-isolated protein samples were then transferred to NC membranes. Subsequently, NC membranes were closed with 5% skim milk for 1 h and then incubated overnight at 4 °C with homologous primary antibodies to GR, CXCR4, CD62L, Ncf1, CitH3, and GADPH (1:1000). The blot was incubated with a secondary antibody for 4 h at 4 °C and coupled with HRP. The proteins were finally visualized with a chemiluminescence detection system.

### qRT-PCR Analysis

Total RNA from cells and healing tissues was extracted using TRIzol. cDNA was reverse-transcribed from RNA to cDNA using Takara RT Master Mix and amplified and detected on a real-time PCR system (StepOne Plus, Applied Biosystems, USA) using 2× SYBR Green qPCR Mix to amplify and detect cDNA targets. Relative mRNA expression fold changes were calculated by using the 2^–ΔΔCt^ method. Primers were synthesized by SeqHealth using the following sequences.

GR-forward: TCCGATGAAGCTTCGGGATG, GR-reverse: AGGTAATTGTGCTGTCCTTCCA; CXCR4-forward: GTGCCAGCCCCTAGATATACAC, CXCR4-reverse: TGCCGACTATGCCAGTCAAG;

CD62L-forward: TGGAGGGCAGAGACAATCCA, CD62L-reverse: GGGATAGGAGCCGTCTAGGG;

Ncf1-forward: GGAGGGCAGAGACAATCCA, Ncf1-reverse: GACGTCAGCTTCCGTTTGGT;

CitH3-forward: ATGAGACCTGGAGAAAAAGCTC, CitH3-reverse: TTACTCICTAACCCACCCCCT; Cyclin D1-forward: CAGCCCCAACAACTTCCTCT,

Cyclin D1-reverse: CAGGGCCTTGACCGGG; Cyclin D3-forward: CCTTCTAAGCTCGCCCTGAA,

Cyclin D3-reverse: GCTCCATCCACTGCCATCATT;

VEGFR-forward: GGAAGGCCCATTGAGTCCAA, VEGFR-reverse: CGATCTGGGGTGGGACATTC.

### EdU Essay

Cell proliferation of HUVECs was detected by the 5-ethyl-2′-deoxyuridine (EdU) doping assay. After treatment, HL-60 cells were washed twice with PBS. HUVECs were stained using the EdU doping method according to the manufacturer’s instructions. EdU-stained images were obtained by using a fluorescence microscope (Olympus, Japan).

### Antibacterial Evaluation of the Hydrogels

*E. coli* and Methicillin-resistant *S. aureus* (MRSA) were used to test the bacteriostatic potential of the EF@S-HGM hydrogel: (1) The bacteria were inoculated by spreading them evenly on the surface of the agar medium. (2) After coculturing the neutrophils with the EF@S-HGM hydrogel, the lower chamber solution was used to spread the bacteria on the medium flatly and incubated at 37 °C for 2 h. The solution was diluted 500-fold and spread evenly on LB agar plates. (3) After incubation at 37 °C for 24 h, pictures were taken with an EPSON V600 digital scanner.

For SEM analysis of the bacteria, a bacterial suspension (100 μL, 108 CFU mL^–1^) was added to the hydrogel samples, and the samples were incubated on the surface of the hydrogel for 3 h. The samples were then analyzed by an NIR laser (80 μL, 108 CFU mL^–1^). NIR laser (808 nm, 1 W cm^–2^) irradiation was then performed for 5 min, followed by sonication for 2 min to separate the bacteria from the hydrogel. After the bacteria were immobilized and dehydrated, they were resuspended in anhydrous ethanol, and 100 μL of the resuspension was added dropwise on the silicon wafer. Subsequently, the bacteria were dried, sputtered with gold, and scanned with a ZEISS Gemini SEM 300 electron microscope from Germany.

### Scratch Assay

HUVECs were seeded into six-well plates. When 90% confluence was reached, the cell monolayer was scratched with a sterile 200 μL microtube tip. Floating cells were washed twice with PBS. HUVECs were subjected to different treatments. Images of each scratch were observed using a light microscope at 0, 12, and 24 h, respectively. Data were analyzed by using ImageJ.

### In Vitro Angiogenesis Assay

Matrigel was first added to a 96-well plate and incubated at 37 °C for 1 h. Cells were then placed into wells coated with Matrigel and divided into groups according to the treatments received. Incubation was performed at 37 °C for 8 h. ImageJ software randomly captured and analyzed three viewpoints for tube lengths and total branching points.

### IHC and IHF Examination

Mice were executed on day 14. Embedding of traumatized tissue was done in paraffin. Samples were frozen and cut into 7 μm-thick slices. They were incubated with anti-CD31 (1:100), anti-GR (1:100), anti-MPO (1:100), and anti-CitH3 (1:100) overnight at 4 °C. Three nonoverlapping high-magnification fields of view were selected and the amount of cd31-positive cells was counted to evaluate the microvessel formation at the wound site.

### Statistical Analysis

All data were calculated using GraphPad Prism 10.1 software, with a *p*-value <0.05 as the significance threshold. Data are presented as mean ± standard deviation (SD). One-way ANOVA was used to analyze group differences, with asterisks indicating significant differences (ns *P* > 0.05, *P < 0.05, **P < 0.01, ***P < 0.001).

## Supporting Information Available

EF@S-HGM hydrogel acting as a wire to make the light bulb glow (MP4)
Rapid gelation ability of the EF@S-HGM hydrogel; compressibility and resilience; shear viscosity curves; stress and strain curves of MeGel, HGM, and EF@S-HGM hydrogels; crystalline violet staining and flow cytometry to detect the apoptosis of neutrophils cultured in hydrogels with different concentrations of ingredients; EdU staining, tube-forming, and scratching experiments after incubation of HUVEC with hydrogels containing different concentrations of components; in vivo treatment of wounds using hydrogels with different concentrations of ingredients; and statistical analysis of the above experiments (PDF).
