# Supplementary material for: Conductive Hydrogel Inspires Neutrophil Extracellular Traps to Combat Bacterial Infections in Wounds
Source: ACS Nano. 2025 Mar 3;19(10):9868–84. doi: 10.1021/acsnano.4c14487 (PMC11924340; doi:10.1021/acsnano.4c14487)
Supplement: Supplementary file 2 — nn4c14487_si_002.pdf [file nn4c14487_si_002.pdf]

Conductive Hydrogel Inspires Neutrophil Extracellular Traps to Combat Bacterial Infections in Wounds

Lizhi OuYang[1#], Ze Lin[1#]. Xi He[23#], Jiaqi Sun[2#], Jiewen Liao[1], Yuheng Liao[1], Xudong Xie[1], Weixian Hu[1], Ruiyin Zeng[1], Ranyang Tao[14], Mengfei Liu[1], Yun Sun[1*], Bobin Mi[1*], Guohui Liu[1*]*

[1] Department of Orthopedics, Union Hospital, Tongji Medical College, Huazhong University of Science and Technology, 1277 Jiefang Avenue, Wuhan, 430022, China.

[2] Union Hospital, Hospital, Tongji Medical College, Huazhong University of Science and Technology, Wuhan, 430030, China.

[3] Department of Rheumatology, Renji Hospital Affiliated to Shanghai Jiao Tong University School of Medicine, Shanghai, 200001, China.

[4] Department of Surgery, Prince of Wales Hospital, The Chinese University of Hong Kong, Hong Kong, 999077, China.

#These authors contributed equally to this work and should be considered co-first authors

*These authors contributed equally to this work and should be considered co-corresponding authors

Correspondence should be sent to Guohui Liu ; e-mail: liuguohui@hust.edu.cn

Supporting Information

1. Supplementary Figure

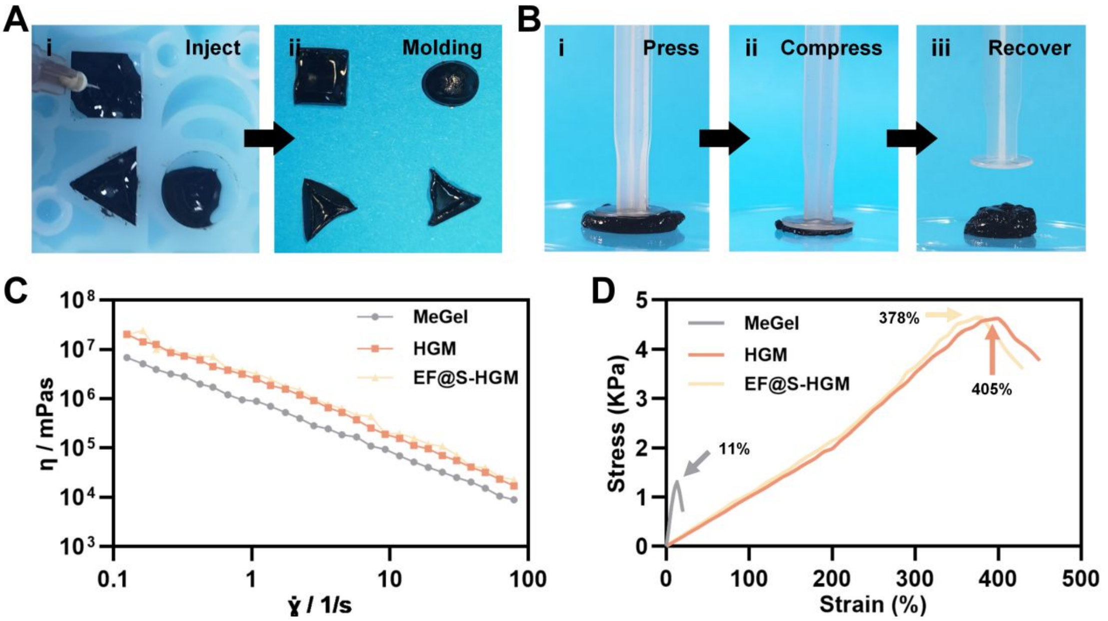

Figure S1. Other physical properties of EF@S-HGM hydrogels. A) EF@S-HGM hydrogel can form a gel quickly on any shape of mold. B) Excellent compressibility and resilience properties. C) Shear viscosity curves of MeGel, HGM, and EF@S-HGM hydrogels, and D) stress and strain curves.

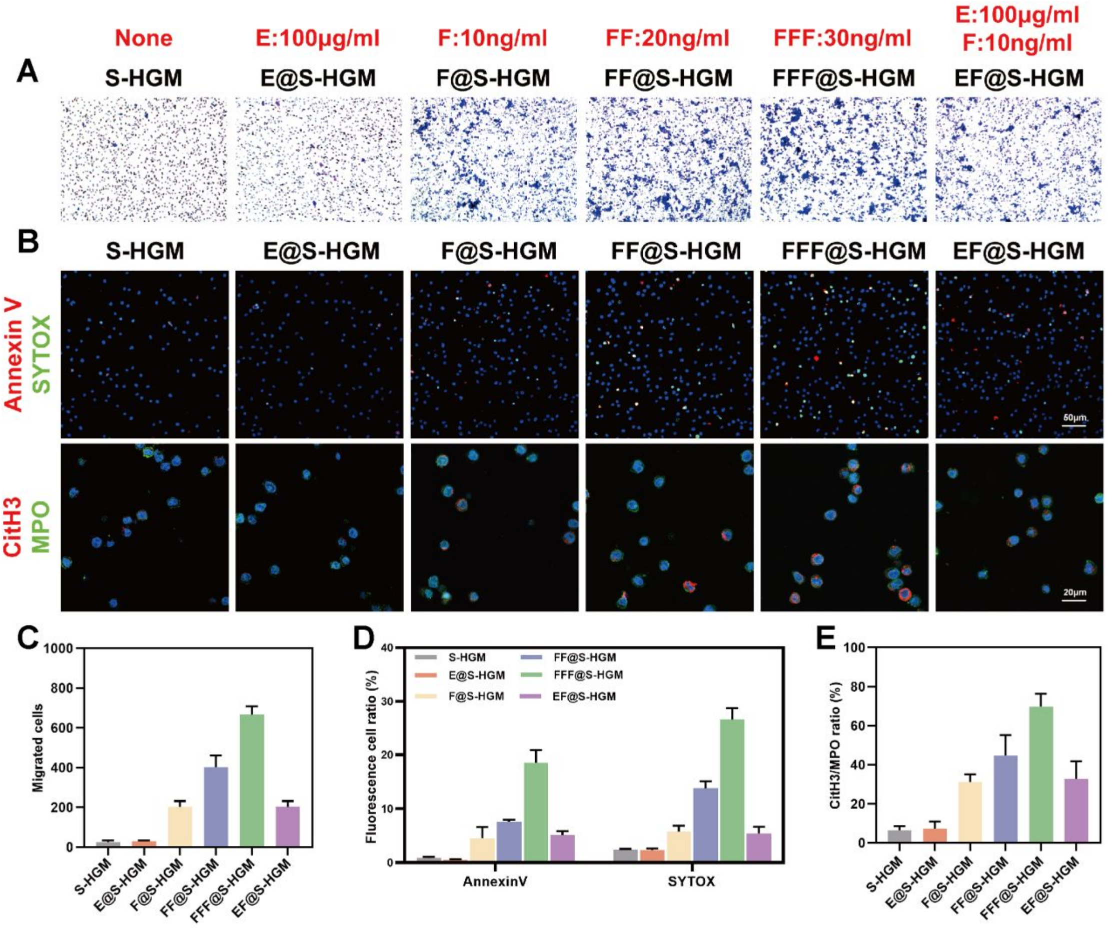

Figure S2. Components in EF@S-HGM hydrogels induce neutrophil migration and NETosis. The drug concentration in each subgroup is shown at the top, where E represents ECGS and F represents FMLP. A) Crystalline violet staining of neutrophils after passing through a Transwell system with a 4 μm pore. C) Quantitative analysis. Scale bar: 100 μm. B) Fluorescence spectra of neutrophils co-cultured with hydrogels. E) Quantitative analysis of the relative ratio of CitH3/MPO. D) Quantitative analysis of the proportion of Annexin V- and SYTOX-double positive cells to total. Scale bars: 100 μm and 20 μm.

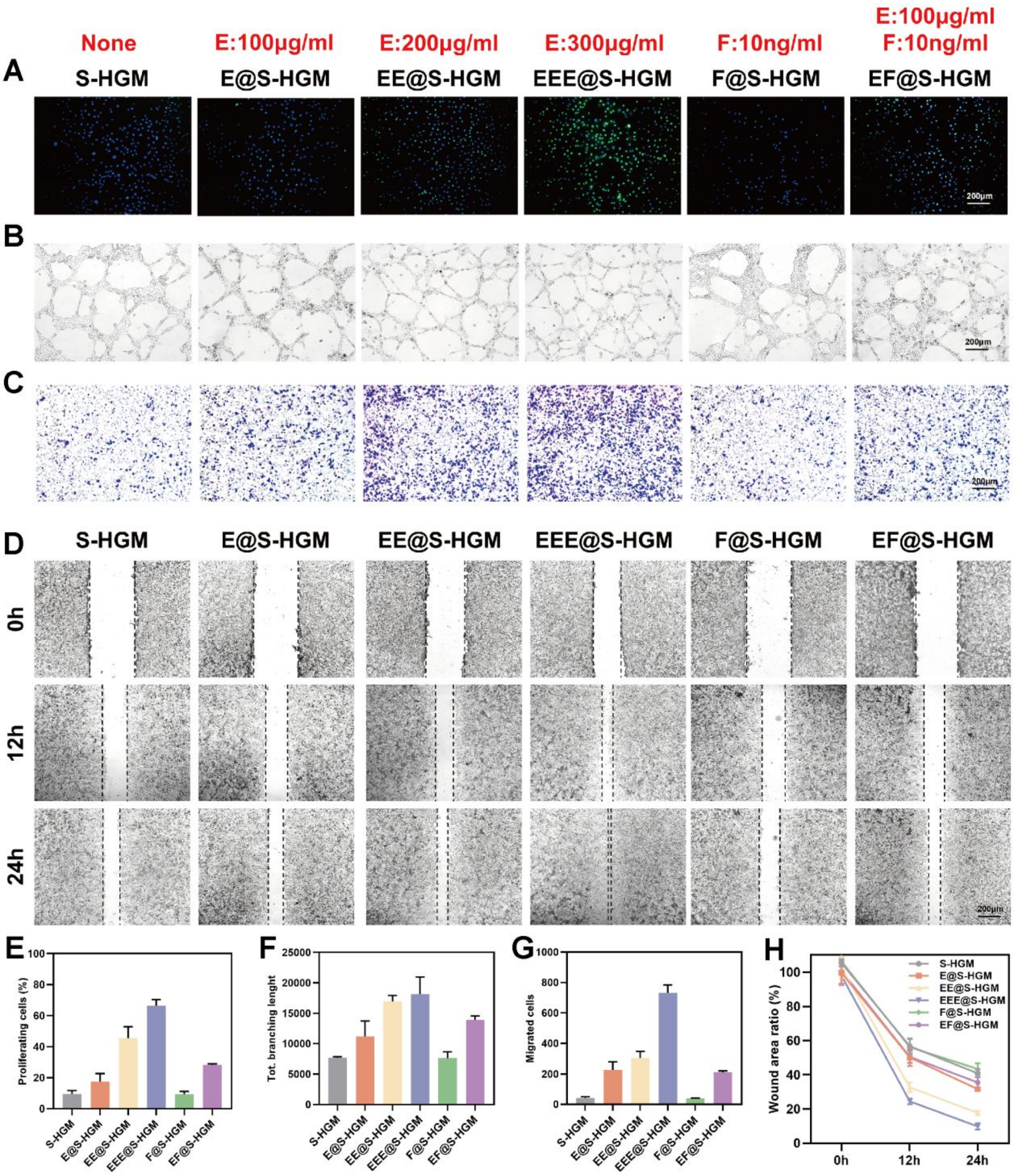

Figure S3. Components in EF@S-HGM hydrogels promote HUVEC repair. The drug concentrations in each subgroup are shown at the top, where E stands for ECGS and F stands for FMLP. A) EdU staining images and E) quantitative analysis of HUVEC after co-culturing with EF@S-HGM hydrogel. Scale bar: 200 μm. B) Tube-forming assay of HUVEC after co-culturation and F) quantitative analysis. Scale bar: 200 μm. C) Transwell crystal violet image of HUVEC after co-culturation and G) quantitative analysis. Scale bar: 200 μm. D) Scratch healing test image of HUVEC and H) quantitative analysis.

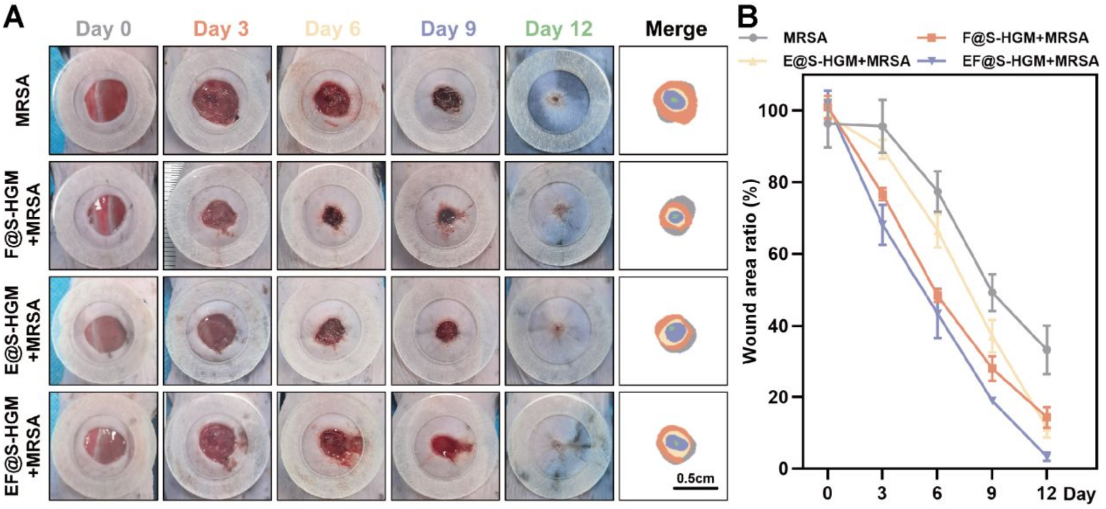

Figure S4. EF@S-HGM hydrogel promote infected wound healing in vivo. Drug concentrations in each subgroup were similar to Figure S2 and S3. A, B) Representative images and statistical analysis of wound closure. Scale bar: 0.5 cm.
